# A new case of lower extremity glomus tumor Up-to date review and case report

**Published:** 2012-06-18

**Authors:** B Frumuseanu, R Balanescu, A Ulici, M Golumbeanu, M Barbu, V Orita, L Topor

**Affiliations:** Department of Pediatric Orthopedics, “Grigore Alexandrescu” Clinical Emergency Hospital for Children, Bucharest

**Keywords:** glomus tumor, glomangioma, glomangiomyoma, reddish nodule

## Abstract

Glomus tumor (glomus cell tumor) is a rare, hamartomatous, usually benign neoplasm, whose cells resemble the modified smooth muscle cells of the normal glomus body.

The diagnosis of a lower extremity is often delayed, due to the lack of awareness and low level of suspicion, by the treating physician. The glomus tumor (GT) often involves the nail beds. The unusual location of the lower extremity often leads to missed or delayed diagnosis and management. There is a paucy of information about GT in general, especially among orthopedic surgeons. The aim of this article is to make the surgical community more aware of this disease

## Case report

A 10-year-old boy presented with a 2-weeks history of tenderness and induration in the medial aspect of the right knee, after a fall on his leg. Physical examination revealed a 5 cm round, mobile mass, well circumscribed, without localized pain to palpation. The patient had no symptomatology before the traumatism.

**Fig. 1:**
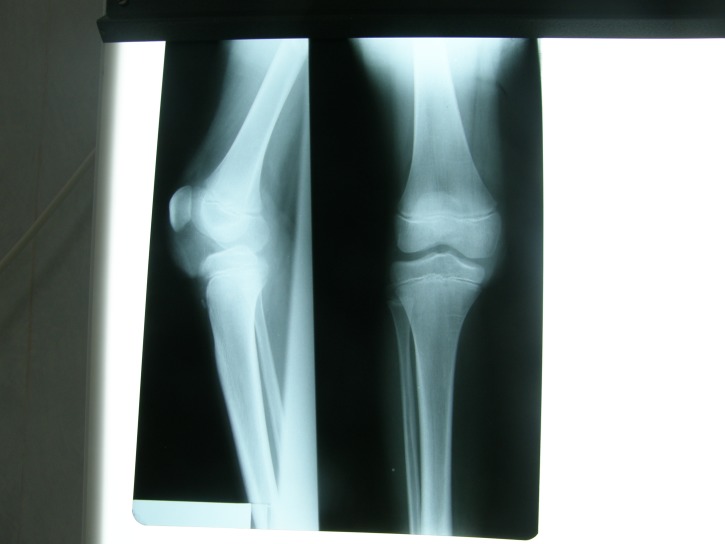
X-ray of the right knee

An X-ray of the right knee and its laboratory values including VSH, CRP, HLG, serum calcium, alkaline phosphatase, phosphorus, magnesium, were normal (**Fig. 1**).

**Fig. 2:**
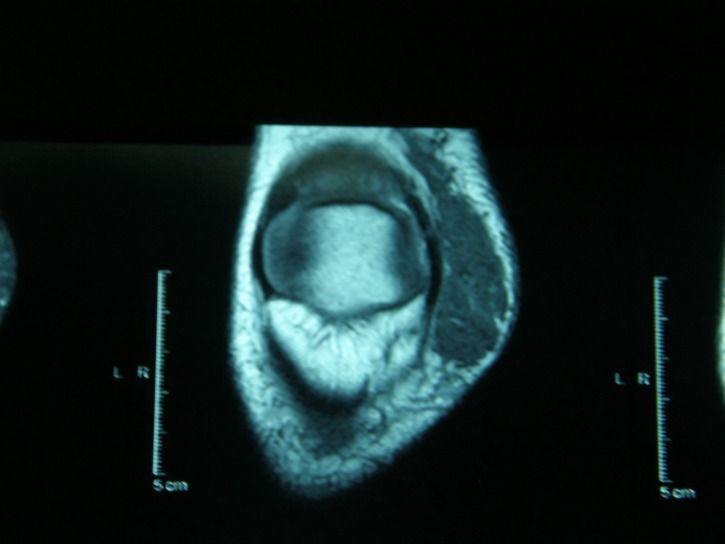
IRM of the right knee - A

**Fig. 3:**
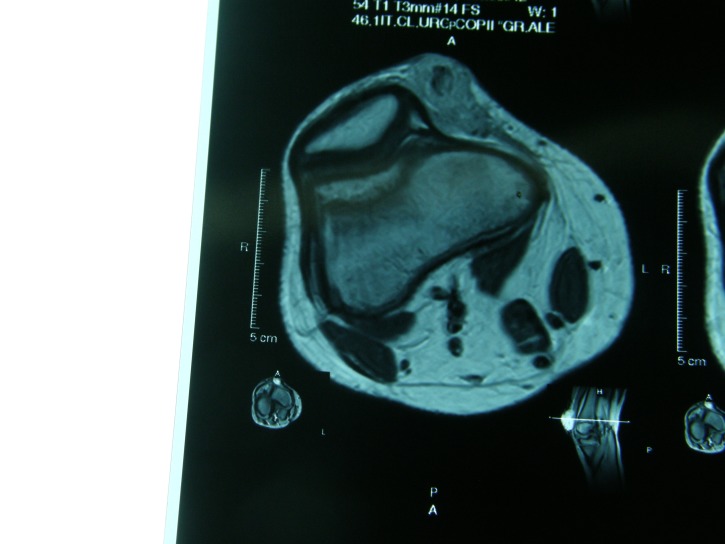
IRM of the right knee - B

**Fig. 4:**
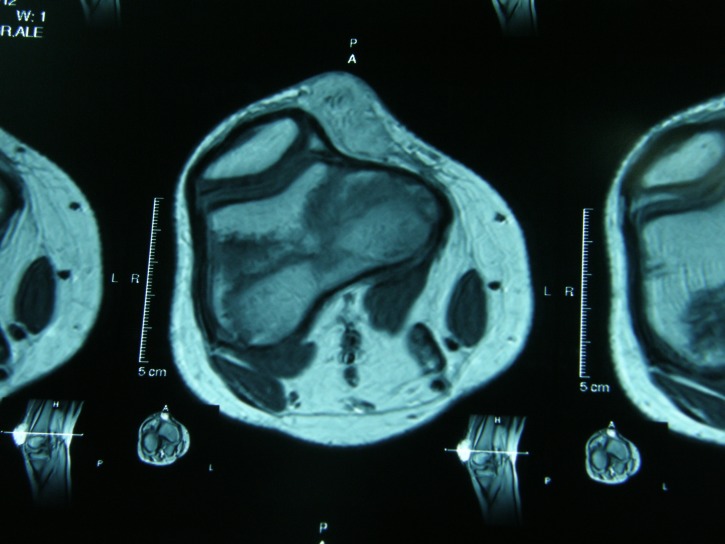
IRM of the right knee – C

The IRM of the right knee revealed an oval soft tissue mass (6,5/3,5/1,5cm) on the medial side of the right knee. The lesion exhibited hypointense signal on MR T1-weighted images, isointense signal on MR T2-weighted images and moderate enhancement after injection of gadolinium (**Fig. 2,3,4**).

The incisional biopsy revealed multiple reddish nodules, well circumscribed. The first histopathology examination established the diagnostic of villonodular synovitis. The second look, in the other department established the diagnostic of glomangyoma.

**Fig. 5:**
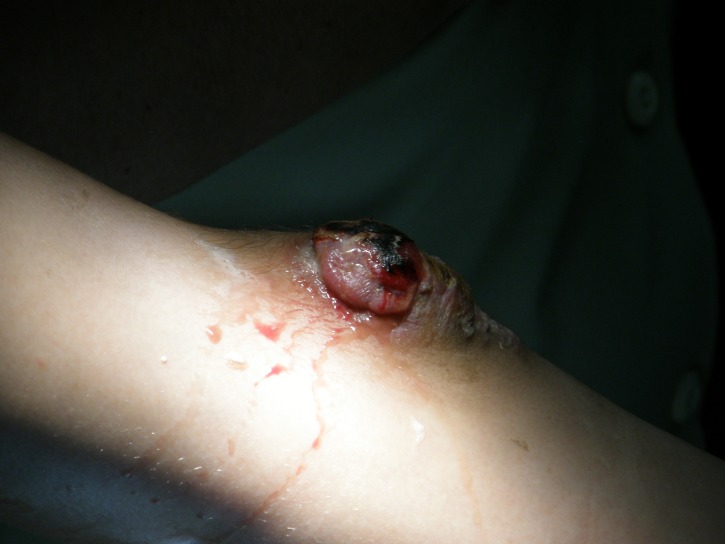
Tumor development

**Fig. 6:**
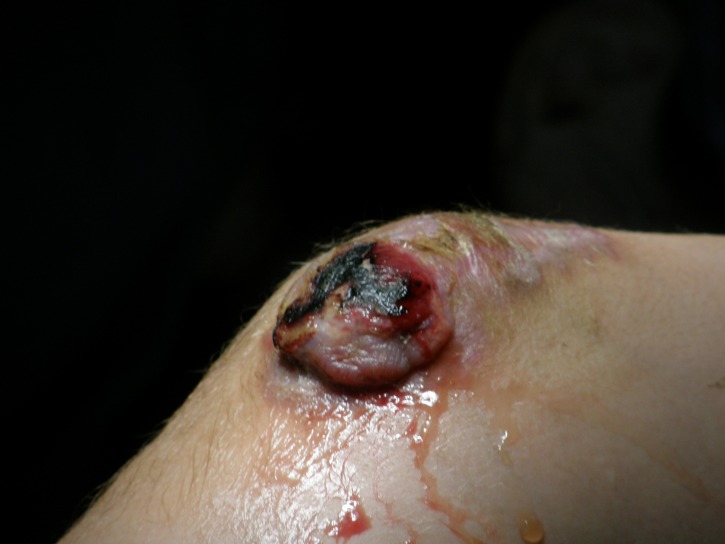
Mushroom-like tumor

In the meantime, the tumor developed outside the skin and became mushroom-like, painless (**Fig. 5,6**). After 2 months, we performed a surgical excision, followed by another histopathological examination. A histopathological diagnosis of glomus tumor was made. The postoperative evolution was very good, without local recurrence after complete excision.

## Discussion

Glomus tumor (GT), also termed tumor of Popoff, or Barre-Masson syndrome, is an extremely rare benign lesion composed of rounded uniform cells often arranged in a brick-work-like manner. They are intimately associated with the vascular structures [**6**]. At one time, this tumor was considered to derive from the neuromyoarterial glomus, a neurovascular structure [**12**]. At present, however, it is believed that the tumor arises from the modified smooth muscle cell. Outside the bone, the glomus tumors with cellular atypia (so-called symplastic glomus tumors) and malignant glomus tumors have been described both of which are exceedingly rare [**8**]. 

Recently, glomuline, a gene located on chromosome 1p21-22 and possibly involved in the differentiation of vascular smooth muscle cells, has been shown to be mutated in familial glomuvenous malformations (glomangiomas) [**4,5**]. Genetic alterations in sporadic glomus tumors have not been published [**7**].

A vast majority of glomus tumors occur at the fingertips [**9**], arising in the distal phalanges [**3**]. The unusual location of the lower extremity often leads to missed or delayed diagnosis and management.

In 1812, Wood made the first clinical description of a GT; he described it as a “painful cutaneous tubercle” [**26**]. In 1924, Masson published its first comprehensive pathologic description [**16**]. These tumors typically present as a painful, firm, purplish, solitary, subcutaneous nodule. Tumor size is generally reported to be small, rarely bigger than 1 cm [**1,2,17**]. When the tumors are located at the lower extremity, the average tumor size is more than 2 cm, in contrast to those tumors located in typical locations (fingers). Symptoms include intense burning pain at the tumor size, which occurs spontaneously or is precipitated by temperature changes or touch. A fear of using the lower extremity may cause severe limb atrophy, extremity vasomotor disturbances [**13**] or flexion restrictions [**14**].

GT has been reported in about 1,6% of 500 consecutive patients with primary soft tissue upper and lower extremity tumors [**11,21**]. Multiple classifications of GT have been proposed by several investigators, depending on the tumor histology and behavior [**8,15,16,19**].

GT may be solitary or multiple; the latter may be further divided into regional or disseminated, which are usually familial or congenital. Other variants, such as plaque type and patch type have been described [**11**]. Solitary GTs are usually seen in adults, commonly equal in both sexes, except for the subungual glomus tumors, which show a female preponderance [**22**]. A solitary glomus tumor is a pink or purple nodule with classic triad of pain, cold sensitivity and point tenderness [**24**]. The commonest site is the hand, particularly the fingers. There have been reports in the literature of unusual location of glomus tumor such as the ankle [**23**], foot [**20**], knee [**2**], thigh [**18**], and hip [**10**].

Histologically, the tumors have variable quantities of glomus cell, blood vessels and smooth muscle [**25**]. Accordingly, they are classified as solid glomus tumor (25%), glomangioma (60%) and glomangiomyoma (15%) [**11**]. Glomangiomyoma is the rarest histological type of the glomus tumor and is frequently located in the lower extremities.

GT has to be differentiated from enchondroma, epidermal inclusion cyst, metastasis, aneurismal bone cyst, sarcoidosis eccrine spiradenoma, leiomyoma, neuromatosis hyperplasia, multiple hemangiomas [**11,12**]. 

The treatment of choice for isolated GT is surgical excision. Sclerotherapy with sodium tetradecyl sulphate, polidocanol and hypertonic saline has been reported to be effective in patients with multiple glomangioma located on the extremities. Ablative therapy with Argon and Carbon dioxide laser is of potential benefit for small, superficial lesions [**11**].

## Conclusion

There is a generalized misconception about the frequency of GT affecting the lower extremities. GT is often misdiagnosed initially and can lead to significant morbidity to the patient owing to severe symptoms, despite its benign appearance. Because of symptoms and malignant potential, diagnosis and treatment of GT are essential. However, owing to its rarity, especially situated in atypical locations, such as the lower extremities, the treating physician must have a high index of suspicion.

## References

[1] Anagnostou  GL, Papadementriou  DG, Toumazani  MN (1973). Subcutaneous glomus tumor. Surg Gynecol Obstet.

[2] Caughey  DE, Highton  TC (1966). Glomus tumour of the knee. Report of a case. J Bone Joint Surg Br.

[3] Bjorkengren AG, Resnick D, Haghighi P (1986). Intraosseous glomus tumor: report of a case and review of the literature. Am J Roentgenol.

[4] Brouillard  P, Boon  LM, Mulliken  JB (2002). Mutations in a novel factor, glomulin, are responsible for glomuvenous malformations (“glomangiomas”). Am J Hum Genet.

[5] Brouillard  P, Ghassibe  M, Penington  A (2005). Four common glomulin mutations cause two thirds of glomuvenous malformations (“familial glomangiomas”): evidence for a founder effect. J Med Genet.

[6] Enzinger  FM, Weiss  SW (1995). Benign tumors and tumorlike lesion of blood vessels. In: Enzinger FM, Weiss SW, eds. Soft tissue tumors, 3rd ed.

[7] Folpe  AL (2002). Glomus tumors. In: Fletcher CDM, Unni KK, Mertens F, eds. World Health Organization classification of tumors. Pathology @ genetics. Tumors of soft tissue and bone.

[8] Folpe  AL, Fanburg-Smith  JC, Miettinen  M (2001). Atypical and malignant glomus tumors: analysis of 52 cases, with a proposal for the reclassification of glomus tumors. Am J Surg Pathol.

[9] Fornage  BD (1988). Glomus tumor in the fingers: diagnosis with US. Radiology.

[10] Gencosmanoglu  R, Inceoglu  R, Kurtkaya-Yapicier O (2010). Glomangioma of the hip. Dermatol Surg.

[11] Ghaneshwar Rao  A, Indira D, Kamal J (2010). Extra digital glomangioma. Indian J Dermatol.

[12] Greenspan  A, Jundt  G, Remagen  W Differential Diagnosis in Orthopaedic Oncology. Second edition.

[13] Heje  M, Bang  C, Jensen  SS (1992). Glomus tumours combing limb hypoplasia. J Bone Joint Surg Br.

[14] Kuru  I, Oktar  SO, Maralcan  G (2005). Familial glomus tumor encountered in the same finger and localization in four family members. Acta Orthop Traumatol Turc.

[15] Liapi-Avgeri G, Karabela-Bouropoulou V, Agnani  N (1994). Glomus tumor. A histological, histochemical and immunohistochemical study of the various types. Pathol Res Pract.

[16] 16. Masson  P (1924). Le glomus neuro-myo-arterial des regions tactile et ses tumours. Lyon Chir.

[17] Mullis  WF, Rosato  FE, Rosato  EF (1972). The glomus tumor. Surg Gynecol Obstet.

[18] Negri  G, Schulte  M, Mohr  W (1997). Glomus tumor with diffuse infiltration of the quadriceps muscle: a case report. Hum Pathol.

[19] Panagiotopoulos  E, Maraziotis  T, Karageorgos  A (2006). A twenty-year delay in diagnosing a glomus knee tumor. Orthopedics.

[20] Quigley  JT (1979). A glomus tumor of the heel pad. A case report. J Bone Joint Surg Am.

[21] Shugart  RR, Soule  EH, Johnson  EW (1963). Glomus Tumor. Surg Gynecol Obstet.

[22] Shivaswamy  KN, Thappa  DM, Jayanthi  S (2003). A solitary painful nodule. Indian J Dermatol Venereol Leprol.

[23] Smyth  M (1971). Glomus-cell tumors in the lower extremity. Report of two cases. J Bone Joint Surg Am.

[24] Walsh  JJ, Eady  JL (2004). Vascular tumors. Hand Clin.

[25] Weedon  D Vascular tumours. In: Skin Pathology. 2nd ed.

[26] Wood  W (1812). On painful subcutaneous tubercle. Edinb med Surg J.

